# Engineered bacteriophage-based bioimaging Technology: Development and applications

**DOI:** 10.1016/j.synbio.2025.07.009

**Published:** 2025-08-06

**Authors:** Yuanzhao Shen, Lichang Sun, Jun Li, Xin Zhou, Ran Wang

**Affiliations:** a Jiangsu Key Laboratory for Food Quality and Safety-State Key Laboratory Cultivation Base of Ministry of Science and Technology, Institute of Food Safety and Nutrition, Jiangsu Academy of Agricultural Sciences, Nanjing, 210014, China; b College of Veterinary Medicine, Institute of Comparative Medicine, Yangzhou University, Yangzhou, China; c Jiangsu Coinnovation Center for Prevention and Control of Important Animal Infectious Diseases and Zoonoses, Yangzhou University, Yangzhou, 225000, China; d Joint International Research Laboratory of Agriculture and Agri-Product Safety, The Ministry of Education of China, Yangzhou University, Yangzhou, 225000, China

**Keywords:** Engineered bacteriophages, Bioimaging, Surface functionalization, Genetic engineering

## Abstract

Engineered bacteriophages (phages) have emerged as powerful and versatile tools for bioimaging, owing to their natural specificity for bacterial targets and their amenability to functional modification. This review summarizes recent advances in the development and application of phage-based imaging probes, with a particular focus on surface functionalization techniques and genetic engineering strategies used to construct functional phage imaging agents. These engineered phage probes have been applied across diverse imaging modalities, including fluorescence, magnetic resonance imaging (MRI), nuclear imaging, near-infrared (NIR) optical imaging, and surface-enhanced Raman scattering (SERS), etc. and have been utilized to enable highly sensitive detection of bacterial pathogens, improved diagnosis of infectious diseases, and monitoring of tissue engineering processes. Despite these innovations, critical challenges remain in ensuring robust target specificity, precise control of labeling stoichiometry, and favorable biocompatibility. Addressing issues such as non-specific probe binding, signal quenching, and immunogenicity will be crucial to fully realize the potential of phage-based bioimaging. Looking ahead, this review discusses future directions for next-generation phage imaging platforms with enhanced specificity, multiplexed functionality, and improved translational potential for clinical diagnostics.

## 1 Introduction

**Fig. 1:**
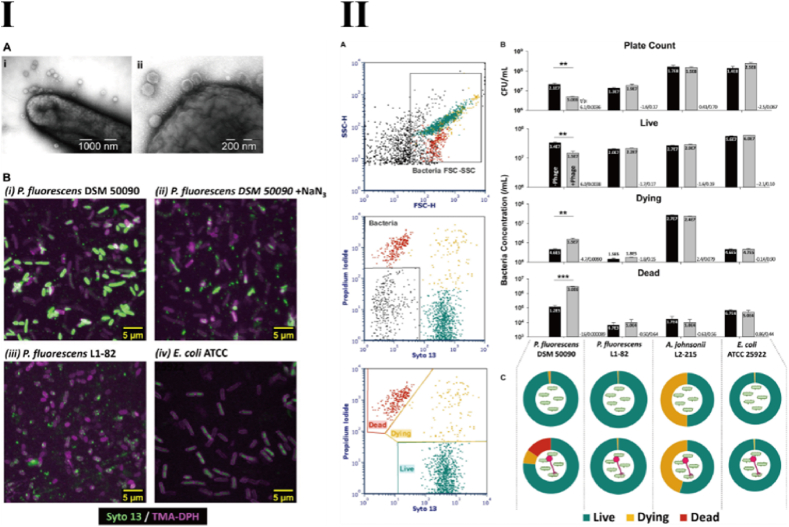
Fast and Easy Phage-Tagging and Live/Dead Analysis for the Rapid Monitoring of Bacteriophage Infection [6]. I: Microscopy of Syto 13 stained PMBT14 binding to and infection of its host. II: Characterization of phage lysis with live/dead cell viability assay by flow cytometry.

**Fig. 2:**
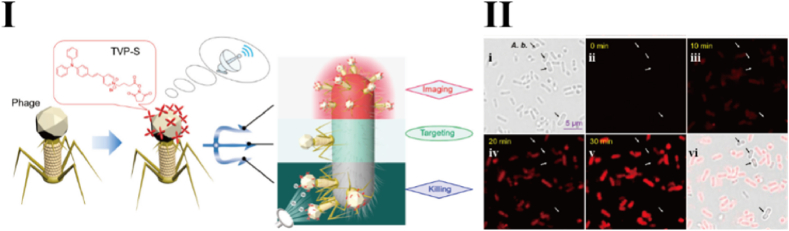
Phage-Guided Targeting, Discriminative Imaging, and Synergistic Killing of Bacteria via AIE Bioconjugates [7]. I: Cartoon illumination of the synergistic effect of AIEgen-modified phage for particularly specific bacterial recognition, real-time fluorescent tracking, phage infection, and AIE PDI activity integrated synergistic bacterial killing. II: Specificity test of TVP-PAP by fluorescence imaging of *P. aeruginosa* and *A. baumanni* (*A.b.* in II i, as an negative control) coincubated with TVP–PAP. Arrows indicate *A. baumannii* without staining by TVP-PAP.

**Fig. 3:**
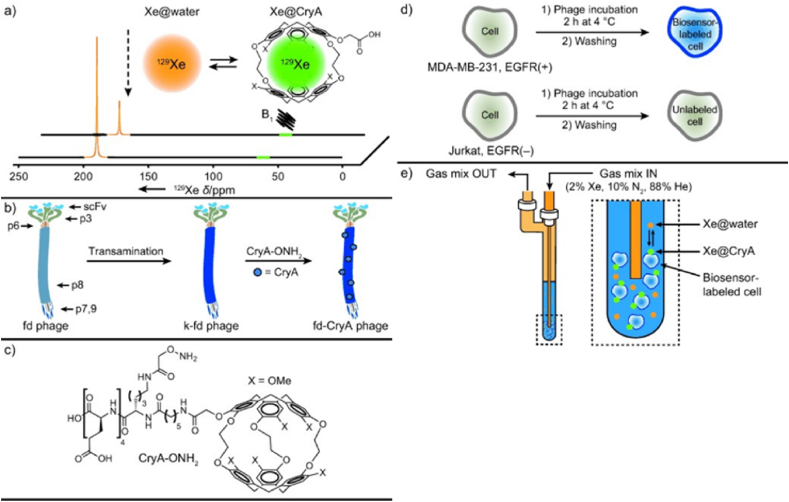
Molecular Imaging of Cancer Cells Using a Bacteriophage-Based ^129^Xe NMR Biosensor [8]. a) The exchange of Xe between solution and CryA cages results in a decrease in the Xe@water signal upon application of a frequency-selective saturation pulse (B1) at the chemical shift of Xe@CryA. b) To modify fd phage with CryA, the phage was first transaminated to introduce ketones at the N-termini of their p8 coat proteins (k-fd) and then incubated with CryA-ONH2 to produce fd-CryA constructs. c) The chemical structure of aminoxy-functionalized cryptophane-A cage (CryA-ONH2). d) For cell-labeling experiments, MDA-MB-231 (EGFR+) and Jurkat (EGFR-) cells were incubated with phage constructs for 2 h at 4 °C. After removing unbound phage, cells were analyzed by flow cytometry, microscopy, or ^129^Xe NMR spectroscopy. e) Live cell Xe NMR experiments were performed in a modified 5 mm NMR tube that allowed for gas flow.

**Fig. 4:**
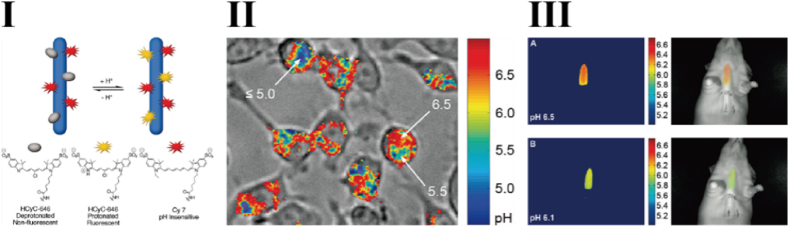
Near Infrared Fluorescence-Based Bacteriophage Particles for Ratiometric pH Imaging [9]. I: Fluorophore Labeled Bacteriophage Particles. II: Intracellular ratiometric pH imaging in RAW cells with the pH-responsive bacteriophage particles. III: Imaging of pH-responsive bacteriophage response in different pH environments.

**Fig. 5:**
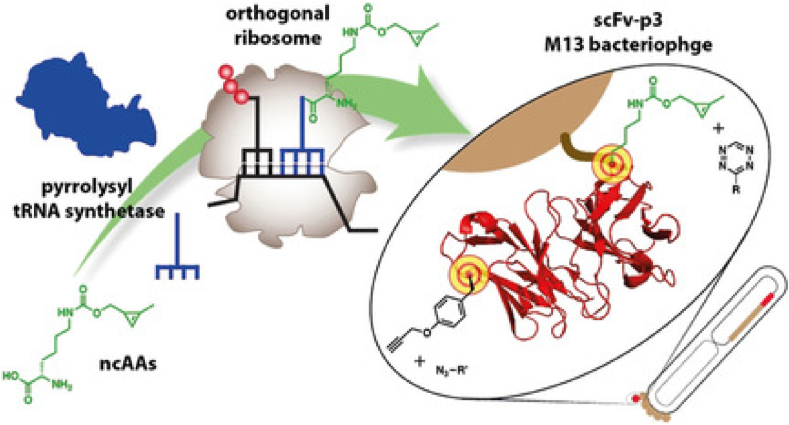
An efficient system for incorporating non-classical amino acids is used to expand the chemical functionality of phage-displayed proteins and the number of encodable new structural units [10].

**Fig. 6:**
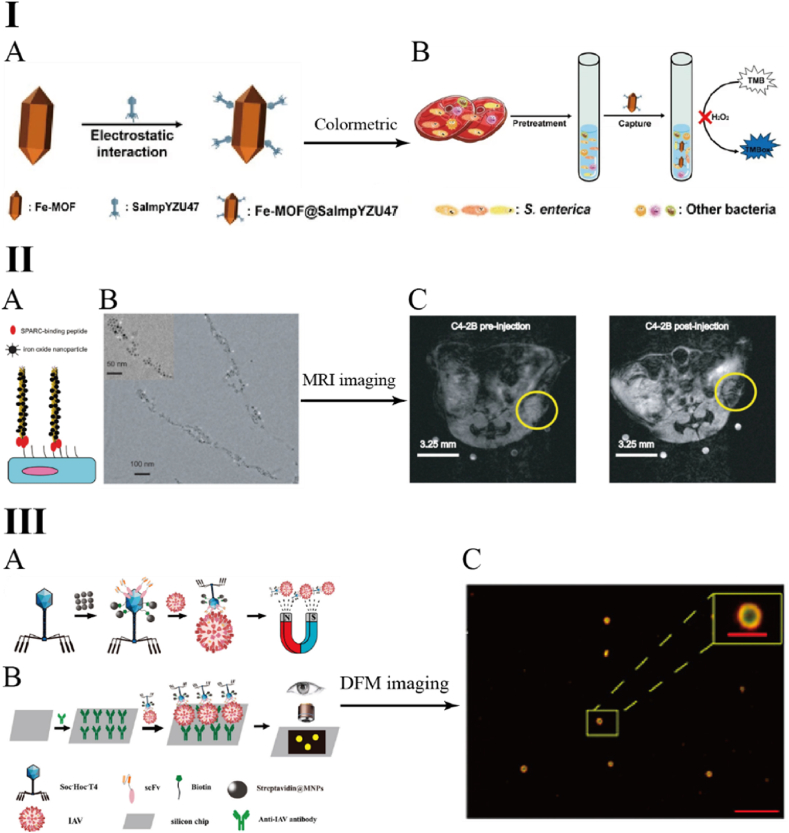
MNP modified phage for colorimetric, MRI and dark-field imaging [11,12,13]. I: Colorimetric quantitative detection of *Salmonella* based on Fe-MOF and phage; II: MRI imaging of mouse tumors based on MNPs and phages; III: Ultrasensitive visual counting strategy for influenza viruses under DFM based on MNP and T4 bacteriophage.

**Fig. 7:**
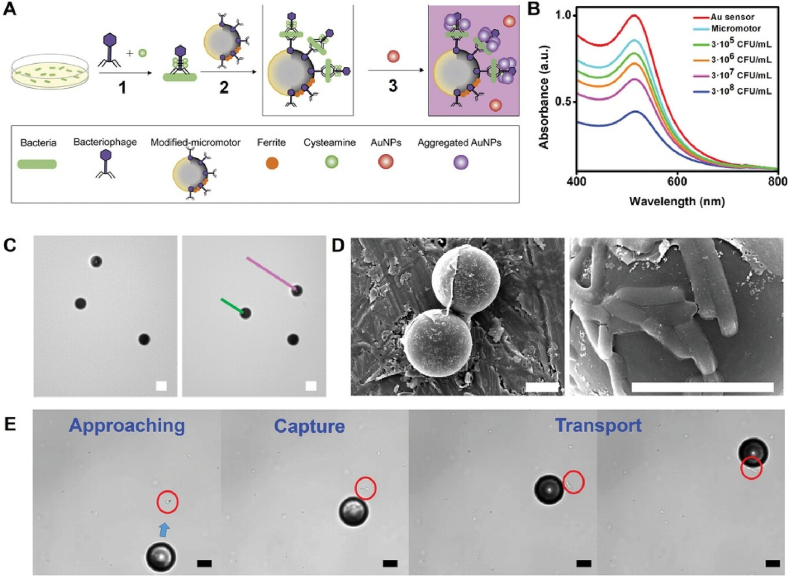
Magnetic Bacteriophage-Engineered Janus Micromotors for Selective Bacteria Capture and Detection [14]. A: Schematic of the bacteriophage-functionalized magnetic Janus micromotors for *E. coli* bacteria biosensing: 1) Generation of the bacteria-cysteamine T4 bacteriophage complex. 2) Incubation of the bacteria-T4 bacteriophage complex with the magnetic phage-modified micromotors. 3) On-the-fly bacteria capture with the micromotors and addition of the AuNPs. The assay was performed in 96 well plates, following measurements with a microplate reader; B: UV–vis spectra of the AuNPs added to the incubated micromotors, with increasing bacteria concentration; C: Time-lapse images illustrating the magnetic motion of the micromotors and corresponding tracking lines; D: Scanning electron microscopy (SEM) images of the micromotors after bacteria capture at two magnifications. E) Time-lapse images showing a micromotor approaching, capturing, and transporting an *E. coli* bacterium. Scale bars, 10 μm.

**Fig. 8:**
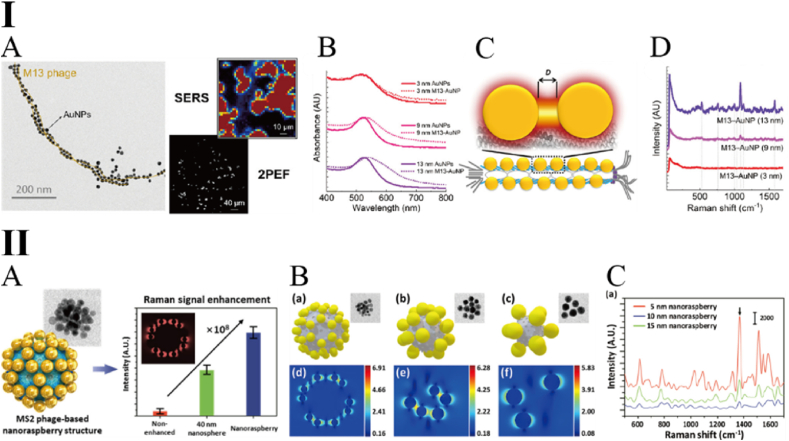
Particle size-mediated SERS performance optimization of AuNP-modified phage [15,16]. I: A: TEM, SERS and 2PEF imaging of AuNP-modified M13 phage; B: UV–vis spectra of AuNPs (solid line) and M13-AuNP assemblies (dashed line); C: Scheme of M13-AuNP assemblies with enhanced localized electric fields. The distance (D) between the adjacent AuNP must be smaller than the particle diameter to observe the coupling. II: A: Schematic diagram of AuNP modified MS2 phage nanoraspberry structure and comparison of SERS signals among MS2 phage, AuNP and nanoraspberry structure; B: Numerical simulation of nanoraspberry structure with variation of nanoparticle size. (a–c) Predicted structure and (inset) representative TEM images. (d–f) Simulated electric field norm at the characteristic xy-plane of nanoraspberry constructed with (a, d) 5 nm, (b, e) 10 nm, and (c, f) 15 nm nanoparticles; C: SERS spectra of rhodamine 6G from nanoraspberry structures with varying sizes of nanoparticles.

**Fig. 9:**
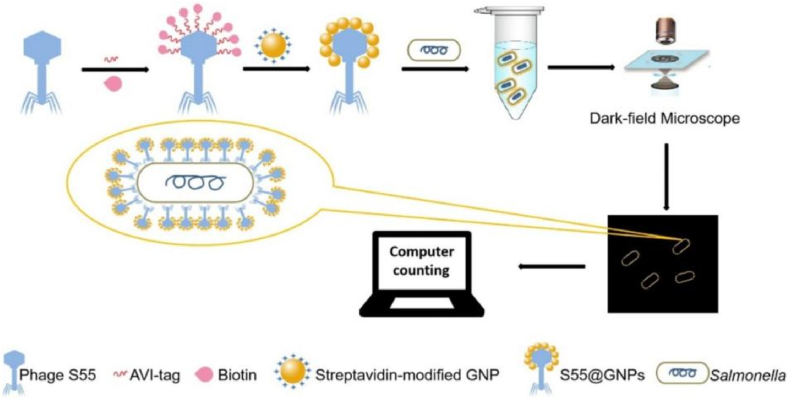
The schematic diagram illustrates the construction of AVI-tag mediated phage@GNPs and subsequent detection of target bacteria under DFM [17]. The Salmonella phage S55 was gene engineered to display an AVI-tag on a specific location of its capsid protein, which was then biotinylated to generate the biotinylated phage. Multiple streptavidin-functionalized GNPs were subsequently attached to the biotinylated phage, resulting in the formation of the S55@GNPs probe. This probe exhibits a distinct golden rod-shaped structure under DFM and selectively binds to target Salmonella, enabling easy enumeration by naked eye and further quantification using counting software.

Bacteriophages (phages) are viruses that specifically infect bacteria bacteria, utilizing the cellular machinery of their hosts to replicate, ultimately lyse the bacteria with high efficiency and specificity [1]. Since the discovery of phages in the early 20th century, phages and antibiotics have been regarded as two major avenues for treating bacterial infections [2]. Beyond their applications in antibacterial therapy, phages also demonstrate significant potential in the field of bioimaging as unique targeting agents [3]. Notably, phage-based imaging probes offer several unique advantages over conventional probes (such as antibodies, nanomaterials, or small-molecule imaging agents). They provide exceptional specificity for bacterial targets and exhibit favorable biocompatibility. Phages are also highly amenable to genetic or chemical modifications, allowing multivalent display of functional molecules on their capsids, and they even have the capacity for self-amplification at target sites. Bioimaging is an essential technology for visualizing biological processes, tracking pathogens, and diagnosing diseases. The development of engineered phage-based bioimaging has progressed from simple modifications of natural phages to advanced genetic engineering techniques. Initially, phages were utilized in bioimaging to explore their biological properties and to create bacterial detection platforms that are highly specific, cost-effective, and easy to prepare [4]. With ongoing advancements in phage research and rapid progress in molecular biology and nanobiotechnology, the highly modifiable and programmable nature of phage capsid proteins allowed them to overcome inherent targeting limitations and function as multifunctional imaging probe carriers. Through surface functionalization and genetic engineering, phages can be modified with a variety of imaging agents, including fluorescent molecules, radioactive isotopes, and nanomaterials, facilitating efficient labeling and imaging of both biological and non-biological targets [5]. Currently, engineered phages have been applied across multiple imaging modalities, including fluorescence imaging, magnetic resonance imaging (MRI), and near-infrared (NIR) imaging, and are widely utilized in areas such as tumor detection, infection site localization, and tissue engineering (see Fig. 1, Fig. 2, Fig. 3, Fig. 4, Fig. 5, Fig. 6, Fig. 7, Fig. 8, Fig. 9).

Although extensive research has demonstrated the broad potential of phages in both *in vivo* and *in vitro* bioimaging applications, significant challenges persist in the strategies used to conjugate imaging probes with phages. Conventional physical or chemical modification techniques often lack precise control over labeling sites and quantities, leading to issues such as non-specific binding, poor structural stability, and considerable variability. These factors, in turn, reduce imaging accuracy and the targeting efficiency of phages. Engineered phages developed through gene editing technologies can display functional peptides or proteins on their capsids, enabling precise, efficient, and specific conjugation with imaging agents. This significantly enhances sensitivity and batch-to-batch consistency while reducing background noise. Moreover, certain gene-edited phages are capable of continuously expressing target proteins across multiple generations, thereby eliminating the need for complex transient modifications and facilitating long-term bioimaging [18]. However, due to the limitations of prokaryotic expression systems and the steric hindrance of capsid proteins, the range of exogenous sequences that can be expressed on phages remains restricted. Additionally, unresolved challenges, including *in vivo* stability, immunogenicity, large-scale production, and standardization, continue to hinder the development of engineered phage-based bioimaging technologies.

In summary, despite promising advancements, the application of engineered phages in bioimaging continues to encounter significant challenges. This review presents a comprehensive summary and analysis of the current state of engineered phage-based bioimaging technologies, emphasizing various modification strategies and their underlying mechanisms. Additionally, it addresses critical bottlenecks in existing applications and provides an initial outlook on future technological developments. We hope this review will provide a robust theoretical foundation and a practical reference for researchers in the field, thereby advancing the development of phage-based bioimaging towards enhanced precision, multifunctionality, efficiency, and safety.

## 2 Phage bioimaging strategies based on surface functionalization

Phages are viruses composed of a genome and structural proteins, such as capsid and tail fiber proteins, which resembling the architecture of conventional viruses. In specific contexts—such as during outbreaks of avian influenza or novel coronavirus—the study of viral distribution dynamics is critical for patient treatment and the development of targeted therapeutics. Therefore, the rapid and accurate quantitative tracking of viruses is of great significance [19,20]. As both “bacterial predators” and “natural nanoparticles”, phages have been widely employed in various imaging studies [21]. Without the need for substantial alterations to their physiological structure, physical or chemical modifications—such as fluorescent labeling or nanomaterial-based conjugation—provide a direct and effective strategy for viral imaging. Many phage-based imaging techniques have adopted similar modification strategies. Accordingly, this section summarizes phage imaging methods based on physical and chemical modifications, analyzes the mechanisms underlying each approach, and discusses their respective advantages and limitations. At the end of this section, to provide a comparative overview of the phage-based imaging strategies, Table 1 summarizes the modification types, signal characteristics, and key advantages and limitations associated with each imaging modality discussed in this section.

**Table 1:** Comparison of representative phage imaging strategies according to imaging modality.

Imaging Modality	Phage Modification Strategy	Key Advantages	Key Limitations
Fluorescent Labeling	Intercalating dyes (e.g., SYBR Gold); Capsid-conjugated dyes (e.g., FITC, Rhodamine)	Simple and rapid labeling; real-time visualization; suitable for microscopy	Low tissue penetration; photobleaching; possible dye leakage or quenching
Radioisotope Labeling	Radiolabeling via bifunctional chelators (e.g., MAG3, DTPA); biotin-avidin bridging	Ultra-sensitive; quantitative *in vivo* imaging; deep penetration	Requires isotope handling; potential radiotoxicity; conjugation complexity
Near-infrared Probe	Covalent coupling of NIR dyes to capsid surface	Better tissue penetration; low background noise; compatible with small-animal imaging	Limited photostability; reduced spatial resolution at greater depths
Nanomaterials	Surface assembly of nanoparticles (e.g., Fe_3_O_4_, AuNP, QD); electrostatic or bioconjugation-based	Enhanced signal intensity; multifunctional readout	Material-dependent toxicity; potential immune clearance; nanoparticle aggregation risks

### 2.1 Fluorescent labeling strategy

As early as 1995, Hennes et al. discovered that fluorescent dyes, such as Yo-Pro-1, could stain the DNA of viruses, including phages, and developed a fluorescence microscopy-based method for virus quantification [22,23]. To achieve adequate staining of the genomic DNA, phages were co-incubated with Yo-Pro-1 in a humid environment for 2 days. Subsequently, Lawrence et al. found that the fluorescent dye YOYO-1 also possessed the ability to stain phage DNA, with an incubation period of up to 72 h [24]. Although prolonged incubation can improve the staining efficiency of fluorescent dyes for phage DNA, it may also lead to nonspecific staining or phage inactivation. To enable rapid staining, Mosier-Boss et al. developed a method using SYBR Gold, which offers more flexible fluorescence excitation conditions and requires only a short incubation time of approximately 10 min. This method also reduces potential interference between the dye and the phage, which could otherwise suppress phage infectivity, thereby yielding clearer imaging results under fluorescence microscopy [25]. Similarly, Deng et al. demonstrated that SYBR Safe, when incubated with phages at 80 °C, can achieve a high level of DNA binding within 10 min [26]. Yang et al. further showed that SYBR Green I can rapidly label the genomic DNA of T7 phages within the same incubation time [27]. These studies indicate that SYBR-based dyes are efficient tools for rapid DNA staining and suggest that their binding affinity to phage DNA may be enhanced under elevated temperature conditions. As the characteristics of various fluorescent dyes continue to be explored, multi-dye labeling of phages holds promise for expanding the functionality of bioimaging. In 2020, Low et al. utilized propidium iodide (PI), a large-molecule fluorescent dye that cannot penetrate intact cell membranes, in combination with Syto 13 to label the *Pseudomonas* phage PMBT14. This dual-staining approach enabled differentiation between live and dead bacteria, thereby broadening the application scope of phage-based bioimaging [6]. Notably, in this study, residual PI and Syto 13 dyes were completely removed by a 0.45 μm PES filter membrane, eliminating the need for centrifugation and avoiding associated damage to the phages, which further improved staining efficiency (Fig. 1).

Although phage staining techniques have continued to improve, DNA-based labeling methods remain inherently unstable. First, non-covalent interactions between dyes and DNA are susceptible to washing, and the dyes may interfere with DNA replication or packaging. Second, the abundance of capsid proteins on the phage surface can further diminish fluorescence intensity. Therefore, DNA-based fluorescent labeling alone does not fully meet the demands of phage-based bioimaging applications. In 2015, Kim et al. utilized the AlexaFluor 555 fluorescent dye-labeled M13 phage as a colorimetric probe for the lateral flow assay (LFA) detection of MS2 phage [28]. In this approach, the abundant P8 capsids on the surface of the M13 phage provide a high density of accessible amino groups, which can react with the carboxyl groups of AlexaFluor 555 through EDC/NHS-mediated condensation. This covalent labeling strategy significantly enhances binding stability, while the high availability of capsid proteins offers numerous reactive sites for dye conjugation, thereby greatly increasing the fluorescence intensity and detection sensitivity of the phages. Building on these advantages, phages can be applied in more complex biological environments, including cellular systems and bodily fluids. For instance, in 2016, Yang et al. conjugated carboxyl-functionalized FITC fluorescent probes to ε-amino groups on the surface of engineered M13 phages targeting non-muscle invasive bladder cancer (NMIBC), enabling targeted imaging of BIU-87 xenograft tumors in mice [29]. The fluorescent phage was injected into the tail vein and significantly enriched at the tumor site within 4–6 h, maintaining a stable signal even after 24 h. Similar conclusions were confirmed in studies conducted by De Plano et al. [30,31]. These findings indicate that FITC-labeled phages, prepared through EDC/NHS-mediated reactions, remain stably bound in systemic circulation and within the complex tumor microenvironment in mice.

Although fluorescent labeling based on capsid proteins offers improved specificity, it remains susceptible to false-positive results due to factors such as inadequate purification and residual non-specific probes. Additionally, conventional fluorescent dyes typically fluoresce only in a monodisperse or free state, while their fluorescence is quenched in aggregated forms, which inevitably limits the achievable dye loading density on phages [32]. In contrast, fluorescent probes that utilize the aggregation-induced emission (AIE) mechanism exhibit the opposite behavior: AIE probes emit strong fluorescence in aggregated or high-concentration states but remain non-emissive when dispersed [33]. Leveraging this unique photophysical property, He et al. developed an AIE-based phage probe for bacteria-specific imaging and synergistic antibacterial activity [7]. In their study, a sulfonic acid-functionalized tetraphenylethylene-vinyl-pyridinium cationic dye (TVP-S) was covalently conjugated to the surface of *Pseudomonas aeruginosa* phage through an amine-carboxyl condensation reaction, resulting in a stable AIE-phage bioconjugate (TVP-PAP). This hybrid probe retains the host specificity of the phage while incorporating the turn-on fluorescence capability of the AIE dye. Notably, TVP-PAP generates a visible fluorescence signal only when it is enriched on the surface of host bacteria, thereby eliminating background interference from free dye and preventing signal quenching caused by excessive modification. This strategy presents a novel approach to enhancing the specificity, sensitivity, and reliability of phage-based imaging technologies (Fig. 2).

It is important to note that while traditional fluorescent probes demonstrate excellent optical properties within the visible light spectrum, and AIE-based probes effectively mitigate issues such as fluorescence quenching due to high-density labeling, their application for *in vivo* imaging remains constrained. This limitation is primarily due to the strong absorption of visible light by endogenous biomolecules, such as hemoglobin and myoglobin, as well as interference from tissue autofluorescence generated by compounds like NADH, FAD, and collagen. Consequently, fluorescence signals in the visible spectrum exhibit limited tissue penetration and high background noise, significantly restricting their effectiveness in deep-tissue or high-contrast *in vivo* imaging applications [34,35].

### 2.2 Radioisotope

Radioisotope imaging technology is a technique that utilizes radioactive tracers (radionuclides) to visualize the distribution of substances within living organisms. The development of this technique has addressed the limitations of traditional fluorescence-based *in vivo* imaging, particularly its low tissue penetration depth and high background noise [36]. Currently, radioisotope imaging technology is widely applied in fields such as medicine, pharmaceutical research, and biology, enabling non-invasive monitoring of physiological, metabolic, and pathological processes [37]. In 2004, Rusckowski et al. were the first to label phages with a radioisotope known as technetium-99 m (^99^ᵐTc), developing an imaging technique for the specific detection of *E. coli* infections *in vivo* in mice [38]. The labeling procedure involved the following steps: first, the carboxyl groups of mercaptoacetyltriglycine (MAG3) were activated through EDC/NHS-mediated reaction, allowing for the formation of stable amide bonds with lysine residues on the surface of the phage. Subsequently, ^99^ᵐTc was coordinated to the thiol group of MAG3, completing the radiolabeling of M13 phages. Experimental results demonstrated that ^99^ᵐTc-labeled M13 phages accumulated specifically at *E. coli* infection sites in mice, where the radioactive signal was significantly higher than in sterile regions. This MAG3-and radioisotope-based labeling strategy was later extended to other phages, including P22, E79, VD-13, and Phage 60, providing a broadly applicable platform for phage-based *in vivo* imaging studies [39].

In a separate study, Holman et al. introduced hydrazinonicotinic acid (HYNIC) as a bifunctional chelator, providing an alternative strategy for radiolabeling phages with technetium-99 m (^99^ᵐTc) [40]. In this method, the *Pseudomonas* phage PAML-31-1 was initially reacted with HYNIC dissolved in dimethylformamide (DMF) for 3 min. During this time, hydrazine group of HYNIC reacted with carbonyl groups on the phage surface to form a stable hydrazone conjugate. This reaction was immediately quenched by the addition of a 0.5 M glycine solution. Subsequently, 115 mM tricine and freshly prepared ^99^ᵐTc were added to the HYNIC–phage solution, along with a SnCl_2_–tricine buffer. SnCl_2_ served to reduce highly oxidized Tc^7+^ to Tc^5+^, which then formed coordination bonds with the pyridine and hydrazine nitrogen atoms of HYNIC, resulting in the construction of formation stable ^99^ᵐTc–HYNIC complex. Experimental validation showed that demonstrated HYNIC-based labeling method achieved both biological activity and labeling efficiency of over 95 %. Compared to the method developed by Rusckowski et al., this approach significantly enhances the efficiency of ^99m^Tc radio-labeling of phages and reduces the overall reaction time, representing a promising new strategy for phage surface modification.

Radioisotope ^129^Xe is recognized as a high-performance probe for *in vivo* imaging. In 2021, Palaniappan et al. developed an ^129^Xe-labeled M13 phage biosensor to facilitate the specific detection and imaging of tumor cell surface molecular markers [8]. In this study, a ketone group was introduced at the N-terminus of the pVIII protein through a transamination reaction catalyzed by pyridoxal-5′-phosphate (PLP). Subsequently, the oxime-modified Cryptophane-A (CryA-ONH_2_) was conjugated to the phage surface, allowing for the capture of ^129^Xe atoms for MRI imaging (Fig. 3). Additionally, a radioisotope-labeled phage, modified with biotin and tagged with N-hydroxysuccinimide-polyethylene glycol-4-biotin (NHS-PEO_4_-biotin), was employed for tumor imaging in a melanoma mouse model, utilizing ^111^In bound to ethylenediaminetetraacetic acid (DTPA)-modified streptavidin [41].

The studies mentioned above demonstrate that phages can be effectively modified using various chemical strategies to facilitate *in vivo* imaging through the incorporation of radioisotopes. The abundance of reactive chemical groups on phage capsid proteins provides numerous binding sites for radiolabeling. Although radioisotope-labeled phages have shown promising results in *in vivo* imaging, high-dose or prolonged exposure to radioisotopes may pose risks of radiation-induced damage to the host organism [42]. It is also important to note that most radioisotopes cannot directly react with the surface chemical groups of phage capsid proteins. Instead, their conjugation often necessitates complex chemical modifications, such as the introduction of multiple functional groups or the use of crosslinkers. These additional steps may compromise binding stability, reduce labeling efficiency, and increase both time and material costs.

### 2.3 Near-infrared probe

Similar to radioisotope imaging, near-infrared (NIR) imaging is also applicable for *in vivo* visualization. NIR light, typically defined as electromagnetic radiation with wavelengths ranging from 700 nm to 1700 nm, offers several advantages, including low tissue autofluorescence, high tissue penetration, and minimal photodamage. These properties have made NIR imaging widely utilized in biomedical applications such as disease diagnosis, molecular imaging, and biomarker detection [43]. In comparison to radioisotope imaging, NIR imaging provides a broader selection of probes and eliminates the use of ionizing radiation. It also facilitates real-time, dynamic imaging with signal acquisition speeds on the millisecond scale. Furthermore, NIR imaging systems offer higher spatial resolution—down to the micrometer or even nanometer level—at relatively lower instrumentation costs, making them particularly suitable for imaging cellular and subcellular structure [[44], [45], [46]].

In 2005, Hilderbrand et al. developed a novel cyanine dye, CyTE-777, and covalently conjugated it to M13 phage capsid proteins through EDC/NHS-mediated reaction, resulting in a stable NIR fluorescent probe for targeted imaging, biosensing, and cellular uptake studies [47]. This method allowed each phage to bind approximately 1700 CyTE-777 molecules without compromising phage viability. To further enhance the functionality of NIR-labeled phages, Hilderbrand et al. established a NIR ratiometric fluorescence imaging platform based on M13 phage phages, employing a similar strategy for precise pH detection in biological systems [9]. In this study, HCyC-646 and Cy7 were NIR dyes with distinct emission spectra. HCyC-646 is pH-sensitive, exhibiting increased fluorescence under acidic conditions. Both NIR dyes (NHS-activated) were conjugated to lysine residues on the phage capsid protein at a defined ratio. With variations in pH, the fluorescence intensity of HCyC-646 changed significantly, while the fluorescence of Cy7 remained constant (Fig. 4). By calculating the ratio of the two fluorescent signals, accurate and quantitative pH measurements could be obtained. This ratiometric strategy is considered suitable for monitoring the acidic tumor microenvironment, disease diagnostics, and other pH-related biological processes.

In addition to the EDC/NHS-mediated reaction, NIR dyes can also be utilized for the direct staining of phages. To investigate the binding behavior between cyanine dyes and phages using this method, Vus et al. evaluated the interaction of five monomethyl cyanine dyes with the MS2 phage [48]. The results indicated that, similar to NIR probes conjugated via the EDC/NHS-mediated reaction, the dyes predominantly bind to the outer capsid of phage rather than its genome. This binding behavior makes them highly sensitive to structural changes of phage, such as thermal or chemical denaturation and variations in salt concentration. For instance, at low salt concentrations (10 mM NaCl), the dye-phage interaction is primarily maintained through electrostatic forces. However, at higher concentrations (150 mM NaCl), electrostatic shielding reduces these diminishes, and hydrophobic allowing become interactions to the rapid and cost-effective nature of the direct staining method makes it more suitable for short-term observation. In observations, if long-term stable fluorescent tracking is required, the EDC/NHS-mediated reaction method offers superior fluorescence stability. Overall, NIR dye labeling of phages is characterized by ease of use, low cost, and favorable biosafety. Nevertheless, like traditional fluorescent dyes, organic NIR dyes suffer from limitations such as poor photostability and significant photobleaching.

To address the issue of non-specificity, Tian et al. developed an M13 phage display system that incorporates noncanonical amino acids (ncAAs) [49]. In this study, unnatural amino acids, such as *p*-acetylphenylalanine, were site-specifically introduced into the pIII capsid of the M13 phage at TAG codon positions. Subsequently, a streptavidin-modified fluorescent probe was covalently attached to the sites of the unnatural amino acid via strain-promoted azide-alkyne [3 + 2] cycloaddition. The results indicated that the binding affinity of the engineered M13 phage for streptavidin increased by approximately 9000-fold. This technique of incorporating exogenous ncAAs into the phage genome is known as genetic code expansion (GCE) technology [50]. In the study conducted by Oller-Salvia et al., GCE was combined with an orthogonal ribosome system to establish a highly efficient phage display platform, enabling dual fluorescent labeling of the M13 phage [10]. Briefly, the cyclopropene-functionalized ncAA (CypK) and the propargyl-functionalized phenylalanine derivative (PrpF) were simultaneously incorporated into the pIII capsid sequence of the M13 (Fig. 5). CypK specifically reacted with tetrazine-modified fluorophores via the Diels-Alder cycloaddition, while PrpF participated in the copper-catalyzed azide-alkyne cycloaddition (CuAAC) to bind with azide-functionalized fluorescent dyes. The results demonstrated that the dual incorporation of these noncanonical amino acids enhanced the labeling efficiency of the M13 phage by up to 10-fold compared to conventional methods. The GCE technology significantly improved the binding capacity between the phage and fluorescent probes while effectively reducing non-specific interactions.

Nonetheless, the fluorescence excitation of dyes and probes can still be influenced by sample preparation procedures, as well as environmental factors such as temperature and pH, and the structural properties of nucleic acids and proteins. Additionally, some dyes have drawbacks including short half-lives and light sensitivity (photoinstability). Prolonged exposure to fluorescent dyes may also negatively impact phage viability. These limitations indicate that while fluorescent dye-based phage labeling can be effective under certain conditions, careful consideration and optimization are necessary during application to enhance detection specificity, sensitivity, and accuracy [[51], [52]].

### 2.4 Nanomaterials

Materials with particle sizes ranging from 1 nm to 100 nm are classified as nanomaterials. Over the past two decades, a wide array of novel nanomaterials has emerged, including those specifically designed for imaging applications [53]. Compared to conventional fluorescent dyes and probes, nanomaterials exhibit significantly enhanced photostability, often resulting in prolonged fluorescence half-live periods. Moreover, the rich modifiability and imaging methods of nanomaterials have expanded their application range and provided the possibility of multiplexing strategies for single materials [54]. It is important to note that nanomaterials lack intrinsic capabilities for the active recognition or capture of bacterial targets. Therefore, to achieve targeted detection, they must be conjugated with bio-recognition elements such as antibodies, proteins, or aptamers that provide specificity. For instance, Cheng et al. functionalized amine-modified magnetic nanoparticles (MNPs) with a high density of *anti*-*E. coli* antibodies to selectively capture *E. coli* from a target sample, achieving quantitative detection of *E. coli* through an ATP-based bioluminescence assay [55]. As a type of live virus that can specifically target host bacteria, phages may outperform small biomolecules in terms of production cost, stability, and modifiability. Consequently, the integration of phages with nanomaterials holds significant promise for bacterial detection applications [56,57]. Phage-nanoparticle conjugates typically utilize the external capsid proteins for functionalization, providing numerous modifiable sites and enabling diverse conjugation strategies. This versatility has further expanded the applicability of phages in bioimaging [58,59].

#### 2.4.1 Magnetic nanomaterials

Among various nanomaterials, MNPs are of particular interest due to their intrinsic imaging capabilities and well-established synthesis and surface modification protocols. Their utility has been extensively demonstrated in the detection of a wide range of biomolecular targets. Magnetic nanomaterials are composed of magnetic elements such as iron, nickel, and cobalt, or their corresponding compounds, and exhibit a diverse array of magnetic properties [60,61]. Depending on the synthesis method employed, the surfaces of these materials can present various functional groups, endowing them with significant surface modifiability. This high degree of chemical versatility makes magnetic nanomaterials particularly suitable for biofunctionalization and targeted imaging applications.

Iron-based magnetic nanomaterials typically exhibit intrinsic peroxidase-like activity, making them suitable for colorimetric detection strategies when conjugated with phages. For instance, Gao et al. employed the EDC/NHS-mediated reaction to immobilize a broad-host-range phage onto an iron-based metal organic framework (Fe-MOF). Utilizing 3,3′,5,5′-tetramethylbenzidine (TMB) as the chromogenic substrate, they successfully developed a colorimetric assay for the detection of *Salmonella* [11]. In the presence of target *Salmonella*, the phages bind to and lyse the bacteria, releasing reductive substances (e.g., glutathione, GSH), which inhibit the catalytic activity of Fe-MOF, resulting in a lighter color in positive samples compared to blank controls (Fig. 6I). Using this approach, Gao et al. successfully detected *Salmonella* in chicken and milk samples, achieving a detection limit as low as 10^3^ CFU/mL. In contrast to iron-based nanomaterials, cobalt-based nanomaterials exhibit superior peroxidase-like activity. In the study by Liu et al., cobalt oxide magnetic nanozymes (Co_3_O_4_ MNEs) were covalently conjugated to specific phage fusion proteins through EDC/NHS-mediated reaction chemistry for the colorimetric detection of *Staphylococcus aureus* [62], achieving a detection limit as low as 8 CFU/mL and demonstrating higher sensitivity than methods based on iron-based nanomaterials.

In addition to their peroxidase-like activity, the superparamagnetic properties of MNPs enable them to exhibit high magnetic susceptibility in the presence of an external magnetic field, making them powerful tools in magnetic resonance imaging (MRI) applications [63]. Leveraging this property, Debadyuti et al. utilized the M13 phage as a scaffold to display cancer cell-targeting ligands and conjugated it with MNPs, facilitating MRI-based visualization of tumors in a mouse model [12]. In this study, monodisperse iron oxide MNPs were assembled along the M13 phage filament through electrostatic interactions with the P8 capsids, whiletumor-targeting SPARC-binding peptides (SBP) were genetically fused to the P3 capsids, resulting in the construction of an engineered phage termed M13-SBP-MNP. The M13-SBP-MNP maintained its structural integrity and demonstrated long-term stability (over 3 weeks) in complex biological environments, including PBS, cell culture medium, and serum, with no observable cytotoxicity. MRI imaging results in a mouse tumor model indicated that, compared to monodisperse SBP-MNP, the relaxation rate of M13-SBP-MNP was reduced by 18 times, and the MRI fluorescence intensity at 1 h and 4 h was 3 times and 11 times that of SBP-MNP, respectively (Fig. 6II). These enhancements significantly improved MRI imaging efficiency and demonstrated strong potential for further advancement in *in vivo* dark-field contrast imaging.

Owing to the unique localized surface plasmon resonance (LSPR) properties, magnetic nanomaterials can be utilized for dark-field microscopy (DFM) imaging without the need for external fluorescent labeling. DFM is an optical contrast technique that blocks direct light and selectively collects light scattered by the specimen, thereby producing bright images of the sample against a dark background [64]. In 2025, He et al. developed a highly sensitive visual counting strategy for influenza virus detection using T4 phage-conjugated MNP and DFM [13]. In this study, streptavidin-modified MNPs were incubated with T4 phages displaying biotin-labeled short peptides (Avi-tags) at 37 °C for 30 min, enabling non-covalent binding through the high-affinity biotin-streptavidin interaction. Under the DFM, T4 phages that successfully captured influenza viruses appeared as distinct “golden yellow fluorescent spots”, and providing high visual contrast and excellent target recognition (Fig. 6III).

In summary, MNPs play a critical role in both target separation and imaging-based tracking. The enzyme-mimicking catalytic activity, MRI contrast enhancement, and LSPR effects of MNP offer significant advantages for multiplexed detection using a single material platform. However, as the core components of MNP are typically chemically reactive compounds, factors such as extreme pH levels, elevated temperatures, biomolecular adsorption, and the presence of ambient magnetic fields may compromise the stability of MNP [[65], [66], [67]]. Consequently, these instabilities can adversely affect detection speed, sensitivity, accuracy, and the overall limit of detection.

#### 2.4.2 Gold nanomaterials

Compared to magnetic nanomaterials, gold nanomaterials demonstrate superior stability and biocompatibility. Due to the enhanced surface plasmon resonance (SPR) effect, gold nanomaterials exhibit exceptional imaging performance in the visible light spectrum, and gold nanomaterials of varying sizes and morphologies can display distinct colors [68,69]. Consequently, the integration of phages with gold nanomaterials has become a widely adopted strategy in bioimaging applications.

Studies have demonstrated that the aggregation of AuNPs induces a shift in their optical absorption wavelength, facilitating substrate-free colorimetric detection [70]. In 2023, Wang et al. developed a visual colorimetric detection method for the rapid and sensitive identification of *Salmonella* with the help of T156 phage and AuNPs [71]. The T156 phage, which contains capsid proteins rich in positively charged amino acids such as lysine and arginine, enabls the electrostatic adsorption of negatively charged AuNPs. When *Salmonella* is introduced, the high affinity of T156 for *Salmonella* surpasses its electrostatic interaction with AuNPs, resulting in the exfoliation of the AuNPs. Under high-salt conditions, the detachment of T156 leads to the loss of the electrostatic stabilization layer, causing the aggregation of AuNPs. This aggregation results in a visible color change of the solution from wine red to purple or blue. Due to this optical property, the assay allows for direct visual detection of *Salmonella* without the need for sophisticated instrumentation.

In the phage-AuNP colorimetric methods described above, the interaction between phages and AuNPs is passive, often resulting in unstable conjugation efficiency. To enhance the binding efficiency between AuNPs and phages, Carmen et al. designed a magnetic phage-engineered Janus micromotor capable of autonomous movement in urine samples for the selective capture and detection of bacteria [14]. In this study, T4 phage was utilized to construct two distinct detection probes. First, Janus micromotors were fabricated by asymmetrically modifying gold-coated polystyrene microparticles with graphene oxide and Fe_2_O_3_ MNPs (Fig. 7). Covalent coupling between the amine groups on the T4 phage capsid and the carboxyl groups on the graphene oxide enabled stable conjugation, forming a magnetically responsive Janus micromotor driven by a weak external magnetic field. In a parallel design, a second type of probe was prepared by modifying the T4 phage with cysteamine to introduce thiol groups for specific functionalization. The detection process was divided into two steps. In the first step, cysteamine-modified T4 phages were added to the urine sample to form a “bacteria-thiolated phage complex”. Subsequently, Janus micromotors functionalized with T4 phages were introduced into the same sample, establishing a sandwich-like structure through binding to the complex. At this stage, monodisperse AuNPs were added, and the thiol groups on the cysteamine facilitated AuNP aggregation within the sandwich structure, resulting in a visible color change in the urine. Quantification of the target bacteria was ultimately achieved by ELISA assay. In this method, the active motion of the Janus micromotor significantly accelerated its interaction with AuNPs, thereby reducing the reaction time. Both the coupling of T4 phages with graphene oxide and with cysteamine was achieved by EDC/NHS-mediated reaction. Considering that the head capsid proteins of T4 phage are rich in amine groups, the functionalization of graphene oxide and cysteamine predominantly occurred at the phage head. This design preserved the integrity of the tail fibers, ensuring that the phage's bacterial capture capability remained unaffected [72].

Studies have demonstrated that the ordered arrangement of AuNPs can exhibit exceptional surface-enhanced Raman scattering (SERS) performance, thereby enabling more sensitive detection [73]. For instance, in 2014, Palanisamy et al. synthesized AuNPs coated with carboxymethyl chitosan (CMC-AuNPs), where the carboxyl groups provided by CMC facilitated specific binding to T7 phages. This interaction promoted the self-assembly of CMC-AuNPs into linear chains along the surface of the T7 phage. The results indicated that, compared to monodispersed CMC-AuNPs, the chain-like arrangement significantly enhanced the optical signal through plasmonic coupling effects, achieving a detection limit as low as 2 fM [74]. Although the phage surface offers abundant modifiable sites, the number of AuNPs that can be bound is inherently limited by spatial constraints and steric hindrance. Therefore, optimizing the size of AuNPs to achieve maximum detection efficiency has become a critical area of research. Conventionally, SERS effects based on AuNP aggregates were believed to occur only with larger nanoparticles (typically >40 nm), while aggregates of smaller AuNPs (<13 nm) were considered insufficient to produce detectable optical enhancement. This limitation significantly restricts the density of AuNPs on phage surfaces and diminishes imaging efficiency [[75], [76], [77]]. Consequently, tailoring the interparticle distance of small-sized AuNPs to ensure the gap between adjacent particles is smaller than their diameter has emerged as a compelling research focus for achieving efficient SERS enhancement [78]. In response to this challenge, Esen et al. successfully demonstrated a SERS effect on M13 phages using small-sized AuNPs. By genetically engineering the pVIII major coat protein to display a gold-binding peptide (GBP), AuNPs with diameters as small as 9 nm were systematically assembled along the filamentous surface of the phage [15]. The results revealed that even such small AuNPs could generate a pronounced SERS signal (Fig. 8I). This study significantly broadened the application scope of AuNP-phage conjugates in nanoscale imaging and detection. Building on a similar strategy, Hou et al. further reduced the size of gold nanoparticles (AuNPs) to 5 nm, enabling the coating of hundreds of AuNPs on the surface of the M13 phage [79]. By utilizing the surface plasmon resonance (SPR) effect of this composite probe, they achieved ultrasensitive quantitative detection of carcinoembryonic antigen (CEA).

It is important to note that, although the pVIII coat protein is widely recognized as the primary site for AuNP functionalization in most studies involving M13 phage, findings by Ahmed et al. revealed a preferential adsorption of AuNPs at the terminal pIII and pIX proteins, rather than along the pVIII backbone [80]. This site-specific interaction was confirmed through both computational simulations and experimental validation, highlighting a previously underestimated binding preference that may inform future probe design strategies. In addition to filamentous phages, Myeong et al. employed an EDC/NHS-mediated reaction to uniformly decorate the surface of the MS2 phage (a member of the Levivirus) with AuNPs measuring 5, 10, and 15 nm in diameter [16]. This highly ordered modification resulted in a significantly enhanced SERS signal compared to that of monodispersed AuNPs, demonstrating the potential of spherical phages as robust scaffolds for SERS-based sensing platforms. In this study, captopril was utilized as a rigid crosslinker, providing thiol and hydroxyl functional groups to facilitate the conjugation of AuNPs and MS2 phages. Theoretically, each MS2 virion could bind up to sixty AuNPs with a diameter of 5 nm, with an average interparticle gap of approximately 1.3 nm. For larger particles, 24 AuNPs (10 nm) and 12 AuNPs (15 nm) could be assembled per virion (Fig. 8II). The resulting AuNP-MS2 conjugates exhibited a deep purple color visible to the naked eye, and their plasmonic absorption peak showed a distinct red shift compared to that of monodispersed AuNPs.

LSPR is one of the fundamental mechanisms underlying SERS. LSPR occurs in the localized regions of metallic nanoparticles, where the enhancement of the local electromagnetic field leads to a significant amplification of the SERS signal from molecules adsorbed on the nanoparticles [81]. Due to the exceptional LSPR properties of AuNPs, phage-AuNP conjugates have found applications not only in colorimetric assays and SERS imaging but also in direct enumeration under DFM. For instance, Masashi et al. developed a rapid quantification strategy for *Staphylococcus* using DFM by co-immobilizing AuNPs and the *Staphylococcus*-specific phage S13′ on the surface of silica (SiO_2_) nanoparticles coated with poly(diallyl dimethyl ammonium chloride) (PDDA) [82]. This multifunctional nanoparticle platform enabled efficient bacterial recognition and produced strong light-scattering signals, facilitating rapid and label-free detection under dark-field conditions. DFM is an ultra-low-background, high-sensitivity imaging technique that provides a more cost-effective alternative to flow cytometry and fluorescence microscopy [83]. Under DFM, the LSPR effect of AuNPs is significantly pronounced, often exceeding the optical emission of even the most potent fluorescent dyes. Consequently, the integration of AuNPs with DFM offers a powerful and economical solution for the rapid and sensitive detection of bacteria. In this study, AuNPs aggregated on the phage capsid produced bright golden spots under DFM, with each spot corresponding to a single *Staphylococcus bacterium*. However, electrostatic interactions are highly sensitive to environmental conditions such as pH, ionic strength, and temperature, making them challenging to maintain in complex biological environments [84]. In 2024, Li et al. utilized a biotin–streptavidin interaction (one of the strongest known non-covalent interactions) to functionalize 15 nm AuNPs on the capsid head of the *Salmonella*-specific phage S55, enabling quantitative detection of *Salmonella* with DFM [17]. In this approach, an Avi-tag (a short peptide containing a single biotinylation site on a lysine residue) was genetically introduced into the head region of S55 phage. Pre-functionalized streptavidin-coated AuNPs were then used for labeling (Fig. 9).

#### 2.4.3 Other nanomaterials

In addition to magnetic and gold nanomaterials, various other metal nanoparticles can exhibit unique imaging mechanisms and conjugation strategies when combined with phages. For instance, Xiao et al. utilized CuO_2_ nanoparticles, which possess oxidative properties akin to those of iron and cobalt nanomaterials, to develop a targeted TMB-based colorimetric assay [85]. The CuO_2_ nanoparticles were conjugated to phages through EDC/NHS-mediated reaction, enabling efficient catalytic oxidation of the TMB substrate and facilitating visually detectable, phage-targeted bacterial detection.

Rare earth elements, including lanthanides, exhibit intrinsic fluorescent properties. Compared to traditional fluorescent dyes and probes, rare earth elements provide several advantages: longer fluorescence lifetimes (ranging from microseconds to milliseconds), enhanced resistance to photobleaching, and narrower emission bands. Utilizing these properties, Yang et al. developed a fluorescent MOF for the quantitative detection of live *Pseudomonas aeruginosa* (PA) by conjugating europium-based nanomaterials (Eu, a representative lanthanide) to PA-specific phages through EDC/NHS-mediated reaction [86].

Quantum dots (QDs) are also widely utilized nanomaterials for fluorescence imaging. Edgar et al. developed a highly sensitive visual detection method for bacteria by coupling biotinylated T7 phages with streptavidin-functionalized CdSe/ZnS quantum dots [87]. Compared to traditional single-element nanoparticles, the CdSe/ZnS core-shell quantum dots provide both tunable emission wavelengths and enhanced photostability, making them well-suited for long-term and multiplexed imaging applications. On one hand, the CdSe core enables precise tuning of the emission wavelength based on particle size, with larger diameters corresponding to longer emission wavelengths. On the other hand, the ZnS shell serves to passivate the surface defect states of CdSe, thereby reducing non-radiative recombination pathways and enhancing the quantum yield. Additionally, the ZnS layer provides a physical barrier that protects the CdSe core from oxidation and photobleaching, significantly improving the photostability of the quantum dots. In comparison to T7 phage, studies have shown that Lambda (λ) phage exhibits a significantly higher binding capacity for QDs, with an average of 13 ± 4 QDs per phage, compared to approximately 2 QDs per T7 phage [88]. This phenomenon may be attributed to the greater structural stability of the λ phage capsid protein gpD [89,90]. Furthermore, T7 is a lytic phage that induces host cell lysis upon infecting *E. coli*, which not only reduces the number of target cells but also generates cellular debris that can interfere with quantum dot conjugation and compromise detection accuracy. In contrast, λ is a temperate phage that does not induce immediate lysis, thereby maintaining a stable environment for phage-cell-QD interactions during the detection process.

In addition to the enhanced imaging sensitivity provided by higher nanoparticle loading, numerous studies have demonstrated that the ordered and dense arrangement of metal nanomaterials can significantly enhance their optical and electrochemical properties. This spatial organization has been shown to greatly amplify signal output, further contributing to the performance of nanomaterial-based detection systems [[91], [92], [93]]. In 2003, Mao et al. introduced a fundamentally different strategy for labeling phages with QDs by utilizing short peptides with a high affinity for metal ions to direct the in situ growth of nanocrystals on the phage surface [94]. In this study, short peptides capable of specifically binding to ZnS or CdS nanocrystals were genetically fused to the pVIII capsid protein, allowing the phage surface to serve as a directional template for the nucleation and growth of ZnS and CdS nanocrystals along the viral filament. By simultaneously displaying two different metal-binding peptides, heterostructured ZnS and CdS nanocrystals were able to co-grow on a single phage scaffold. The authors attributed this phenomenon to the helical structure and highly ordered arrangement of the pVIII capsids, which form a natural linear template. Given that M13 is a filamentous phage approximately 800 nm in length, its modification efficiency is likely to surpass that of λ phage.

Certain non-metallic nanomaterials also possess excellent optical properties and have been utilized in phage-based imaging studies. For instance, Bardhan et al. developed an *in vivo* NIR fluorescence imaging probe by functionalizing single-walled carbon nanotubes (SWNTs) with M13 phages, utilizing the photoluminescence (PL) properties of SWNTs [95]. In this study, the conjugation between SWNTs and M13 phages was based on a fundamentally different mechanism: π-π interactions between the pVIII coat proteins of the phage and the surface of the SWNTs. π-π stacking is a type of non-covalent interaction that typically occurs between aromatic rings containing conjugated π-electron systems. The pVIII proteins of the M13 phage are rich in aromatic amino acids such as tryptophan, tyrosine, and phenylalanine. These residues facilitate π–π interactions between their aromatic rings and the π-electron system on the surface of SWNTs, facilitating non-covalent phage labeling. However, typical π–π interactions are relatively weak compared to hydrogen or ionic bonds, and their binding is often dynamic and reversible. Variations in solvent polarity, pH, or temperature can disrupt π–π stacking, leading to structural instability [96]. Therefore, when utilizing π–π interactions for phage labeling, it is critical to carefully consider the subsequent environmental conditions or to combine these interactions with hydrogen bonding or electrostatic interactions to enhance binding stability [97].

Silicon (Si)-based nanomaterials represent a significant class of non-metallic fluorescent nanomaterials with considerable potential for deep-tissue imaging. In the study conducted by Laura et al., a novel imaging strategy was developed by generating silicon nanoparticles (SiNPs) through pulsed laser ablation in liquids (PLAL), directly within a phage-containing buffer solution [98]. The resulting SiNP-phage conjugates were utilized for the specific recognition of peripheral blood mononuclear cells (PBMCs), demonstrating the effectiveness of Si-based platforms in targeted biomedical detection. PLAL can be applied to the synthesis of nanoparticles from a diverse range of materials, including gold, silver, magnesium, and zinc, and it enables the direct conjugation of nanomaterials with biomolecules [99,100]. Nanomaterials synthesized via PLAL possess abundant surface hydroxyl and silanol groups, which facilitate direct conjugation with phages during synthesis. This process allows for “one-step rapid functionalization” eliminating the need for additional modification steps [101].

In summary, phage-based bioimaging technologies achieved through the surface functionalization of nanomaterials, have been widely applied and have significantly advanced research in this field. The primary advantages of these approaches lie in their technological maturity and operational flexibility. Nearly all types of nanomaterials can be coupled with phages through physical or chemical conjugations. By leveraging differences in material properties and spatial arrangements, diverse imaging modalities can be realized, ranging from *in vitro* to *in vivo* applications and from visible to non-visible spectral ranges. However, these technologies also present certain limitations. Most nanomaterials, in addition to their optical properties, possess other physicochemical characteristics that may adversely affect phage activity and infectivity. For instance, silver nanoparticles have been reported to interfere with the phage lytic cycle by disrupting replication processes, thereby reducing infection efficiency [102]. Additionally, CdSe quantum dots may release cytotoxic Cd^2+^ ions under specific conditions and generate reactive oxygen species (ROS) upon light exposure, potentially inducing oxidative stress responses [103]. In addition, surface functionalization methods typically lack specificity, making it challenging to precisely control the conjugation sites and the number of attached molecules. This can lead to significant batch-to-batch variability and may alter the surface properties of phages during the labeling process, thereby interfering with their interactions with host cells. Furthermore, to minimize interference from non-specific binding, repeated purification steps are often necessary for both the phage and the imaging probe. These additional steps not only reduce the overall efficiency of probe fabrication but may also compromise the native functionality of the phage.

To address the limitations of non-specific labeling, studies conducted by Tian, Li, and Mao introduced exogenous non-canonical amino acids and short peptides into phage coat proteins through genetic engineering [49,17,94]. These studies primarily employed genetic engineering techniques to incorporate exogenous biomolecules into phages. Genetic editing enables precise modifications of phage coat proteins, allowing for the site-specific fusion of tag peptides, fluorescent proteins, or binding domains. This method facilitates the creation of engineered phages with uniform and controllable labeling sites, which are genetically encoded and suitable for scalable production. It can be concluded that gene editing technologies have already begun to transform, or are on the verge of transforming the existing paradigm of engineered phage-based bioimaging.

## 3 Phage-based bioimaging technologies enabled by genetic engineering

Gene editing technologies, such as CRISPR/Cas, enable precise modifications of the genome. In phages, these techniques can be used to integrate one or more exogenous sequences, allowing for the simultaneous expression of diverse functional peptides or proteins. This integration facilitates the combination of imaging capabilities with other functional modules, such as gene knockout or transcriptional regulation, thereby facilitating multimodal research. Gene editing not only minimizes the impact of non-specific modifications by enabling the intrinsic integration of imaging functionalities but also overcomes key limitations in achieving long-term and stable imaging. Consequently, it offers innovative design strategies for the development of advanced phage-based bioimaging technologies [104].

Current research has clearly demonstrated that phages can be genetically engineered to express fluorescent proteins by modifying tolerant or non-essential coat proteins. However, due to the inherent size constraints and steric hindrance of the phage structure, key challenges remain unresolved, specifically regarding how to enhance the display efficiency of exogenous biomolecules and how to effectively express larger molecular weight proteins on the phage surface. M13, T7, and T4 are commonly used model phages for genetic engineering applications. Among these, the M13 phage is the most widely utilized and highly amenable to genetic modification. Its pIII and pVIII coat proteins serve as the primary fusion sites for the expression of exogenous peptides and proteins [105]. Due to its terminal position on the phage and minimal steric hindrance, the pIII protein is well-suited for displaying large molecular weight proteins, despite its low expression level of approximately 3–5 copies per phage particle. In contrast, pVIII is the major coat protein of the M13 phage, with a high copy number of approximately 2700 per particle; however, it is typically limited to presenting short peptide sequences. Display technologies based on the T7 phage rely on the gp10B coat protein, which is present at approximately 415 copies per phage particle. Studies have demonstrated that the C-terminus of gp10B can accommodate peptides or proteins ranging from 50 to 1200 amino acids in length. The expression efficiency is closely related to the molecular size and structural complexity of the inserted sequence [106]. On the surface of the T4 phage, the small outer capsid protein (Soc) and the highly immunogenic outer capsid protein (Hoc) are considered non-essential for the phage assembly. Hoc, present at approximately 155 copies per particle, is well-suited for displaying larger proteins, while Soc, with around 810 copies, is more appropriate for presenting small proteins or peptides [107]. In addition to T4, genetic engineering techniques have also been extensively reported for other phages, such as MS2, λ, and Qβ.

Phage-based bioimaging strategies enabled by genetic engineering can be broadly categorized into three main approaches. The first approach involves displaying exogenous biomolecules on the phage surface to facilitate specific binding with imaging probes, thereby endowing the phage with imaging functionality. For instance, in 2023, Farkas et al. developed a PET imaging probe based on the MS2 phage and the radioisotope ^64^Cu [108]. Through genetic engineering, researchers introduced two functional sites into the coat protein of the MS2 phage: the N87C site for thiol-specific modification and the T19paF site for outer surface functionalization. The N87C site was conjugated with DOTA via a maleimide-thiol reaction, forming a DOTA-MS2 complex capable of chelating metal ions. This modification enabled the labeling of ^64^Cu, facilitating its application in PET imaging. Although this strategy enhances the binding capacity and efficiency of the phage to some extent, it does not completely eliminate the potential effects of non-specific interactions. In contrast, the second strategy effectively circumvents the issue of non-specific binding. As an example of this strategy, Hoang et al. developed a phage-based imaging method by inserting the gene encoding cytochrome *c* peroxidase (CCP), a chromogenic enzyme, into the genome of the wild-type PP01 phage, thereby constructing a recombinant PP01-ccp phage. Upon specifically infecting *E. coli* O157:H7, the PP01-ccp phage expresses the CCP enzyme, facilitating rapid colorimetric detection of low concentrations of *E. coli* O157:H7 within a short timeframe [109]. Since CCP does not produce any luminescent signal in the absence of its substrates, this strategy demonstrates minimal background noise and effectively avoids non-specific binding. Similarly, Schofield et al. genetically engineered the *Yersinia* phage ϕA1122 to encode the luminescent gene cassette *luxAB*. Upon addition of the substrate solution containing decanal, a bioluminescent signal was detected. In summary, this strategy involves phages that encode exogenous proteins, which are neither inherently luminescent nor conjugated to any fluorescent materials or biomolecules. Imaging necessitates the external addition of chromogenic or luminescent substrates. Since these substrates are absent in test samples, the system avoids any potential non-specific luminescence. However, it is important to note that the requirement for exogenous substrates significantly limits the applicability of this strategy in *in vivo* imaging studies.

Considering the advantages and limitations of the two strategies mentioned above, the third approach involves the direct fusion of luminescent biomolecules to phage coat proteins, thereby endowing them with intrinsic visualizability. For instance, Oda et al. genetically modified the Soc coat protein of phage PP01 to express a fusion with green fluorescent protein (GFP), resulting in an engineered phage capable of emitting intrinsic green fluorescence [110]. The results indicated that the fusion of GFP with the Soc protein did not alter the host range of phage PP01. On the contrary, it even enhanced the affinity of phage toward its bacterial host. The engineered phage was ultimately applied for the detection of *E. coli* O157:H7 under a fluorescence microscope. Building upon this work, Raheela et al. discovered that the gene e of phage PP01 encodes an endolysin responsible for host cell lysis. By inserting the GFP gene into gene e and simultaneously terminating its expression, they constructed an engineered lysis-deficient phage, designated “PP01e-/GFP” [111]. This recombinant phage retains the ability to adsorb *E. coli* O157:H7 but does not lyse the host cells. Consequently, as the viable host bacteria replicate, the PP01e-/GFP phages also amplify, leading to an increase in fluorescence signal. By monitoring changes in fluorescence intensity, this system enables rapid discrimination between live and dead bacteria.

Phage-based bioimaging technologies enabled by genetic engineering offer significant advantages, particularly in terms of specificity and functional integrity. By utilizing gene editing tools such as CRISPR-Cas technology, fluorescent protein genes can be directly integrated into the phage genome, making fluorescence labeling an intrinsic property of the phage. Unlike physical or chemical labeling methods, this approach avoids interference with the native biological functions of the phages. For instance, fluorescent labeling can be achieved through the natural gene expression machinery of phage, without compromising its efficiency in infecting host cells. Moreover, gene editing significantly enhances the specificity and accuracy of bioimaging. By precisely targeting the insertion site within the phage genome, fluorescent signals can be ensured to originate exclusively from the engineered phage, rather than from host cells or environmental contaminants. This highly specific labeling strategy not only improves the signal-to-noise ratio but also enables dynamic monitoring of phage behavior in complex biological environments. For example, during the infection process within host cells, fluorescent proteins expressed by genetically labeled phages can report their infection pathways and spatial distribution in real time, eliminating the need for additional fluorescent probes. Compared to chemical labeling methods, which are prone to photobleaching, fluorescence signals generated through genetic editing are more stable and thus well-suited for long-term tracking studies.

Despite the numerous advantages, phage genetic engineering strategies still require further optimization. For instance, the selection of fluorescent proteins must strike a balance between optical properties and expression efficiency. Additionally, in certain complex environments, the gene editing process may be constrained by the genetic background or exogenous selection pressures of the host cell. Therefore, future research should aim to integrate the strengths of both genetic engineering and conventional chemical labeling to develop more efficient and robust bioimaging strategies. For instance, the endogenous expression of fluorescent proteins through gene editing could be combined with nanomaterials to enhance fluorescence intensity or paired with chemical labeling techniques to improve optical sensitivity. To consolidate the diverse genetic strategies described above, Table 2 summarizes the key methods, phage types, application scenarios, and associated advantages and limitations.

**Table 2:** Summary of genetic engineering strategies in phage-based bioimaging.

Genetic Strategy	Target/Labeling Type	Application Scenario	Advantages	Limitations
Fusion of Fluorescent Proteins	Fluorescent reporter expressed on phage capsid or in host	Detection of target bacteria; phage tracking *in vitro*/*in vivo*	Stable, heritable labeling; zero-background signal; real-time infection reporting	Limited brightness; restricted to shallow imaging; expression level depends on insertion site
Display of Binding Peptides/Tags	Peptides genetically inserted into coat proteins for conjugation	Facilitated surface labeling with fluorophores, nanoparticles, or enzymes	Precise site-specific modification; high reproducibility; minimal off-target labeling	Tag size and position may affect phage infectivity or stability
Genetically Encoded Enzyme Display	Reporter enzymes fused to capsid proteins	In vivo bioluminescence imaging of infected cells or tissues	High sensitivity; enzyme signal amplification; substrate-based specificity	Requires external substrate; possible immune recognition of non-native enzymes
Expression of Biorthogonal Reactive Groups	Non-canonical amino acid incorporation for site-specific chemistry	Selective fluorescent or nanoparticle labeling using click chemistry	Unnatural residues enable high-specificity conjugation; avoids background	Requires optimized genetic code expansion system; lower expression efficiency
Genome Replacement/Lytic Gene Editing	Insert reporter genes in place of lytic genes to create non-lytic, signal-producing phages	Bacterial viability-based imaging (fluorescence only from viable infected bacteria)	Signal tightly linked to target viability; avoids killing bacteria before detection	May reduce phage infectivity or propagation; engineering is complex

## 4 Conclusions and perspectives

With the increasing prominence of phages as natural nanobiocarriers in the biomedical field, engineered phage-based bioimaging technologies are emerging as innovative platforms for pathogen detection, disease diagnosis, and drug delivery. The unique advantages of phages, including their intrinsic nanoscale dimensions, structural stability, ease of modification, host specificity, and efficient amplification, demonstrate their significant potential in bioimaging technology. Indeed, compared to conventional imaging probes such as antibodies, nanomaterials, or small-molecule imaging agents, phage-based imaging agents exhibit superior targeting specificity, greater engineering flexibility, improved biocompatibility, and high scalability in production. For instance, unlike monoclonal antibodies, which are complex to produce and inherently limited to bivalent binding, phages can be mass-produced in bacterial cultures and display multiple binding ligands per virion, greatly enhancing target avidity. Similarly, whereas synthetic nanomaterials or small-molecule imaging agents often lack intrinsic targeting ability and must be conjugated with exogenous recognition molecules, phages inherently recognize specific bacterial hosts and can be genetically retargeted to new markers, offering a level of specificity and versatility unattainable by most conventional probes. As biological entities, phages are typically non-toxic to mammalian cells and naturally degrade after use, thereby avoiding the toxicity or clearance issues associated with some inorganic nanoparticles. Notably, phages also possess the unique capacity for self-amplification in the presence of host bacteria, whereby infection and replication at the target site can amplify the imaging signal—a mechanism beyond the capabilities of static probes. These characteristics illustrate the multifaceted advantages of phage-based bioimaging and underscore its value as a complementary or alternative strategy to traditional probe-based imaging in various applications. This review systematically summarizes the current advancements in engineered phage imaging technologies, with a particular emphasis on various labeling strategies and their biomedical applications.

In the early stages, phage-based bioimaging primarily relied on the non-specific binding of fluorescent dyes to nucleic acids or capsid proteins. Although these methods were simple to implement and widely used, they suffered from several limitations, including prolonged labeling times, high background noise, severe photobleaching, and poor stability. To address these challenges, researchers have developed more precise protein-based chemical modification strategies, such as site-specific labeling of amino, carboxyl, and tyrosine residues, as well as non-canonical amino acids on the phage capsid. These advancements have significantly improved labeling efficiency and specificity, enabling stable imaging of phages in complex biological environments, such as the tumor microenvironment and bodily fluids. However, even with these chemical modification strategies, issues such as non-specific binding and batch-to-batch variability persist, underscoring the need for further optimization to enhance accuracy and reproducibility in practical applications.

In recent years, the rapid advancement of nanomaterial technologies has led to transformative progress in phage-based bioimaging. Magnetic nanoparticles, AuNPs, QDs, and other multifunctional nanomaterials have significantly expanded the application scope of phage imaging due to their stable optical and magnetic properties, as well as their excellent biocompatibility. For instance, the integration of magnetic nanoparticles not only accelerates bacterial separation and detection but also serves as a valuable tool for MRI. AuNPs, through LSPR and SERS effects, substantially enhance imaging sensitivity and specificity. Despite these promising outcomes, challenges such as material toxicity, non-specific adsorption, and long-term stability must still be thoroughly evaluated and addressed, particularly in scenarios involving long-term or *in vivo* applications.

To further enhance the precision and efficiency of phage labeling, genetically engineered phage strategies based on gene editing technologies have garnered increasing attention. These approaches involve the targeted modification of phage capsid proteins to display functional peptides or proteins at specific sites, allowing for precise control over labeling locations, high batch-to-batch consistency, and improved labeling efficiency. For instance, the fusion of specific binding peptides or non-canonical amino acids into capsid proteins enables phages to bind fluorescent probes, radioisotopes, and nanomaterials with high specificity and efficiency. This not only overcomes the non-specific binding issues associated with chemical labeling methods but also positions phages as advanced platforms for integrated imaging and therapeutic applications. However, several critical challenges remain, including the low display efficiency of large exogenous proteins, a limited host range, and potential disruptions to capsid structural stability. These issues require urgent further investigation and technological innovation.

Engineered phage-based bioimaging technologies are gradually transitioning from laboratory research to preclinical applications, providing innovative approaches for disease diagnosis, pathogen detection, drug development, and tumor-targeted therapy. Future directions may include: (1) Development of safer and more efficient labeling strategies, particularly those utilizing biocompatible nanomaterials and naturally derived markers such as fluorescent proteins; (2) Integration of advanced gene editing technologies with AI-assisted design to optimize phage capsid structures and enhance the efficiency of exogenous protein display; (3) Construction of multifunctional phage platforms capable of combining imaging, diagnosis, and therapy for real-time, multimodal monitoring and precision treatment; and (4) Deeper understanding of phage-host interactions to improve host specificity and reduce potential toxicity and immunogenicity, thereby accelerating clinical translation. In conclusion, although engineered phage-based bioimaging technologies still face numerous challenges, their remarkable advantages and promising prospects should not be overlooked. With ongoing advances in fundamental research and technological innovation, this field is poised to become a powerful tool in precision medicine, biomedical diagnostics, and therapeutic applications. We hope that this review will provide researchers with a comprehensive understanding of the current landscape of engineered phage imaging technologies and offer valuable insights and inspiration for future innovations in the field.

## CRediT authorship contribution statement

**Yuanzhao Shen:** Writing – original draft. **Lichang Sun:** Writing – original draft. **Jun Li:** Writing – original draft. **Xin Zhou:** Writing – review & editing. **Ran Wang:** Writing – review & editing.

## Conflict of interest

The authors declare that they have no known competing financial interests or personal relationships that could have appeared to influence the work reported in this paper.
